# Antimicrobial Therapy as a Risk Factor of Multidrug-Resistant *Acinetobacter* Infection in COVID-19 Patients Admitted to the Intensive Care Unit

**DOI:** 10.1155/2023/4951273

**Published:** 2023-09-14

**Authors:** P. Mihalov, J. Hodosy, A. Koščálová, M. Čaprnda, M. Kachlíková, J. Jurenka, M. Bendžala, P. Sabaka

**Affiliations:** ^1^Department of Infectology and Geographical Medicine, Faculty of Medicine, Comenius University in Bratislava, Bratislava, Slovakia; ^2^Emergency Department, University Hospital in Bratislava, Bratislava, Slovakia; ^3^Institute of Molecular Biomedicine, Faculty of Medicine, Comenius University in Bratislava, Bratislava, Slovakia; ^4^1st Department of Internal Medicine, Faculty of Medicine, Comenius University in Bratislava, Bratislava, Slovakia

## Abstract

**Background:**

Multidrug-resistant *Acinetobacter* (MDR-Ab) is one of the most important pathogens causing superinfections in COVID-19 patients hospitalised in the intensive care unit (ICU). The occurrence of MDR-Ab superinfection significantly impairs the prognosis of patients in the ICU. Overuse of antibiotics in COVID-19 patients might contribute to the risk of developing MDR-Ab infection.

**Objective:**

The objective was to assess the role of prior antibiotic exposure as an independent predictor of MDR-Ab infection in COVID-19 patients admitted to the ICU.

**Methods:**

We conducted a retrospective cohort study in 90 patients admitted to the ICU of the Department of Infectology and Geographical Medicine, University Hospital in Bratislava, for respiratory failure due to COVID-19 between 1 September 2021 and 31 January 2022 (delta variant predominance). Patients underwent regular microbial screening. Superinfection was defined as infection occurring ≥48 h after admission. We assessed the role of prior antibiotic exposure and other factors as independent predictors of MDR-Ab isolation.

**Results:**

Fifty-eight male and 32 female patients were included in the analysis. Multidrug-resistant bacteria were cultured in 43 patients (47.8%), and MDR-Ab was isolated in 37 patients. Thirty-three (36.7%) patients had superinfection caused by MDR-Ab. Fifty-four (60%) patients were exposed to antibiotics prior to MDR-Ab isolation; of those, 35 (64.8%) patients received ceftriaxone. Prior exposure to ceftriaxone (odds ratio (OR) 4.1; 95% confidence interval (CI) 1.4–11.9; *P* < 0.05), tocilizumab therapy (OR 4.7; 95% CI 1.3–15.0; *P* < 0.05), and ICU length of stay exceeding 11 days (OR 3.7; 95% CI 1.3–10.3; *P* < 0.05) were independent predictors of MDR-Ab infection.

**Conclusions:**

Prior exposure to ceftriaxone increases the risk of MDR-Ab infection in COVID-19 patients admitted to the ICU. Our findings suggest that antibiotic use in COVID-19 patients admitted to the ICU should be restricted to patients with documented bacterial superinfection.

## 1. Introduction

Coronavirus disease 2019 (COVID-19) is caused by the novel severe acute respiratory syndrome coronavirus 2 (SARS-CoV-2). In the vast majority of cases, it is a self-limiting disease. Nevertheless, the infection is able to cause interstitial pneumonia, acute respiratory distress syndrome (ARDS), and hypoxemic respiratory failure. Patients with ARDS often require high-flow nasal oxygen (HFNO) or mechanical ventilation and admission to the intensive care unit (ICU) [1]. While bacterial or fungal superinfections in COVID-19 patients are rare on presentation to the hospital, they are more common among patients admitted to the ICU [2–4]. Superinfections in COVID-19 are associated with a higher intubation rate and increased in-hospital mortality and ICU length of stay [5–7]. Some of the most important pathogens causing superinfections in COVID-19 patients in the ICU are multidrug-resistant *Acinetobacter* strains (MDR-Ab) [8, 9]. MDR-Ab may cause outbreaks in ICU and non-ICU wards; they are difficult to control by standard epidemiological measures [10–13]. The ability of *Acinetobacter* spp. to cause outbreaks in the ICU setting is based on its ability to survive on various surfaces under dry conditions and its resistance to antimicrobials and standard decontamination procedures [14, 15]. The incidence of MDR-Ab superinfections in the ICU was already high in the prepandemic era, and it has increased during COVID-19 pandemic due to the strain on health care systems and the wide usage of broad-spectrum antibiotics and immunosuppressants [15–18]. Opportunistic MDR-Ab infections are further facilitated by immune dysregulation associated with severe COVID-19 [19, 20]. MDR-Ab superinfections in COVID-19 are associated with worse patient outcomes and greater use of resources [7, 21–23]. Empiric antimicrobial therapy is abundantly prescribed in COVID-19 patients with no evidence of bacterial superinfection, while current guidelines do not specifically address this issue [18, 24, 25]. Some of the antimicrobials have been linked to the risk of superinfections by multidrug-resistant bacteria in COVID-19 patients; however, the evidence is still relatively weak [26, 27]. Overuse of antibiotics in COVID-19 patients is potentially manageable. Therefore, we focused on whether exposure to the most frequently used antibiotics increases the risk of MDR-Ab superinfection in patients with COVID-19 admitted to the ICU.

## 2. Methods

We conducted a retrospective cohort study to determine possible risk factors of MDR-Ab isolation and infection in the COVID-19 patients admitted to the ICU. We enrolled all patients meeting the inclusion criteria admitted to the ICU of the Department of Infectology and Geographical Medicine, University Hospital in Bratislava, between 1 September 2021 and 31 January 2022 (delta variant predominance in our region). The inclusion criteria were COVID-19 infection confirmed by polymerase chain reaction for SARS-CoV-2 RNA from the nasopharyngeal swab at the time of admission or a maximum of 14 days preceding admission, acute hypoxemic respiratory failure determined by oxygen saturation below 90% on ambient air, and ICU length of stay of at least 48 hours.

All patients underwent regular microbiological screening during their ICU stay. Sputum or tracheal aspirate, oropharyngeal swab, nasopharyngeal swab, and urine for cultivation were collected three times per week. Blood sampling for galactomannan examination for the presence of invasive aspergillosis was conducted weekly. Sampling for blood cultures was conducted in case of new onset of fever, suspicion of sepsis, or central venous catheter infection.

The following patient data were collected from the hospital information system: demographics; history of diabetes mellitus; clinical data such as need for oxygen therapy (HFNO and mechanical ventilation), antimicrobial treatment, immunomodulatory treatment, development of bacterial pneumonia, ventilator-associated pneumonia, sepsis, urinary tract infection, and central venous catheter-associated bloodstream infection; microbiological data; ICU length of stay; in-hospital death; any infection during hospitalisation; duration of antibiotic therapy; procedures (insertion of central venous catheter, orotracheal intubation, continuous renal replacement therapy); and anamnestic MDR-Ab colonisation or clinical infection during hospitalisation. Empiric antimicrobial treatment was defined as any treatment with systemic bacteriostatic or bactericidal antibiotics started during hospitalisation prior to identification of pathogens of possible superinfection. The diagnosed infections were classified as hospital acquired if their first clinical presentation developed at least 48 hours after admission. Infections in patients with COVID-19 were defined as superinfection if diagnosis occurred ≥48 hours after admission for COVID-19. Only superinfections were included in this study. The types of superinfections were defined and classified according to the CDC/NHSN Surveillance Definitions for Specific Types of Infections [28].

Isolation and identification of *Acinetobacter* strains and other bacteria were conducted according to local laboratory techniques using blood agar cultivation and matrix-assisted laser desorption/ionisation (MALDI-TOF, MBT Smart, Bruker Daltonics Inc., Billerica, MA, USA). Minimal inhibitory concentrations (MICs) of isolated bacterial strains were assessed using an in-house broth microdilution assay. MICs were established according to the European Committee on Antimicrobial Susceptibility Testing (EUCAST) breakpoints [29]. Acinetobacter species isolates and other isolated bacteria were considered MDR in concordance with international expert proposals for interim standard definitions for acquired resistance [30].

Quantitative variables are expressed as medians and interquartile ranges. According to the Kolmogorov–Smirnov test, the quantitative variables in our cohort were not normally distributed. Hence, medians of quantitative variables were compared between groups using the Mann–Whitney *U* test. Receiver operating characteristic curve analysis was used to determine the optimal cut-off value for ICU length of stay associated with risk of MDR-Ab development. Associations between MDR-Ab isolation and infection and the selected variables were assessed using a multivariate binary logistic regression model with a forward stepwise procedure, entering all variables with *P* < 0.1 in univariate analysis. Statistical significance was established at *P* < 0.05. All reported *P* values are two-tailed. The odds ratio (OR) and 95% confidence intervals (CIs) were used to quantify the strength of the associations between covariates and dependent variables. SPSS version 20 (IBM Corp., Armonk, NY, USA) was used for statistical analysis.

This study was carried out in accordance with the Code of Ethics of the World Medical Association (Declaration of Helsinki) for experiments involving humans and was approved by the local Ethical Committee of University Hospital in Bratislava. Written informed consent for participation was obtained from all participants before enrolment in the study. No administrative permission to access the raw data used in this study was required by local authorities or the University Hospital. The raw data were fully anonymised before use. We have preserved the full anonymity of all participants.

## 3. Results

Our analysis included 90 patients (58 males and 32 females). Thirty-eight (42.2%) patients died during hospitalisation. The remaining 52 (57.8%) patients were discharged from hospital after stabilisation in the ICU and full recovery in the non-ICU COVID-19 ward. Forty-two (46.7%) patients were intubated and mechanically ventilated. Thirty-eight (42.2%) patients were treated with HFNO and 10 (11.1%) patients were treated with conventional oxygen therapy.

Forty-three (47.8%) patients were colonised or infected by MDR bacteria; Table 1 provides the ADR isolates. Most of the MDR isolates belonged to the *Acinetobacter* genus. Thirty-three patients (36.7%) had superinfection caused by MDR-Ab. MDR-Ab was the most prevalent cause of MDR bacterial superinfection (76.7% of patients with MDR bacterial superinfection). *Acinetobacter baumannii* was isolated in 22 cases, and all isolates except two were carbapenem resistant. In the remaining cases, *Acinetobacter junii*, *Acinetobacter johnsonii*, *Acinetobacter dijkshoorniae*, or *Acinetobacter pittii* was isolated. All these isolates were carbapenem susceptible but met the criteria for MDR pathogens. The antimicrobial resistance pattern of *Acinetobacter* isolates is provided in Table 2. The most common MDR-Ab superinfection was ventilator-associated pneumonia in 16 patients, followed by pneumonia not associated with mechanical ventilation in 10 patients. Four patients had an MDR-Ab bloodstream infection, and three had a urinary tract infection. Four patients with MDR-Ab isolates did not meet the criteria for superinfection and were regarded as colonisation. Fifty-four (60%) patients were exposed to antibiotic therapy during hospital stay. Ceftriaxone was used in 35 (38.9%) patients, which means that 64.8% of patients exposed to antibiotics received ceftriaxone. Meropenem was the second most utilised antibiotic used in 15 (16.7%) patients. Two patients were treated with cefoperazone-sulbactam, one with moxifloxacin and one with azithromycin.

In 42 (45.6%) patients, the ICU length of stay exceeded 11 days. The baseline patient characteristics are provided in Tables 3 and 4. Patients with MDR-Ab isolates had a significantly longer ICU length of stay (Table 2). In univariate analysis, ICU length of stay, exposure to ceftriaxone and tocilizumab, and mechanical ventilation were significantly associated with MDR-Ab isolation (Table 4). In the multivariate analysis, previous exposure to ceftriaxone (OR 4.1, 95% CI 1.4–11.9, *P* < 0.05), tocilizumab therapy (OR 4.7, 95% CI 1.3–15.0, *P* < 0.05), and ICU length of stay exceeding 11 days (OR 3.707, 95% CI 1.332–10.317, *P* < * *0.05) were positively associated with the risk of MDR-Ab superinfection. The observed trend of increased risk of MDR-Ab isolation in patients on mechanical ventilation was not statistically significant (Table 5). Of 37 patients with MDR-Ab isolates, 17 (51.5%) died compared with 21 (36.8%) deaths in patients without MDR-Ab superinfection. However, the observed trend was not significant.

We created a simple scoring system to stratify the risk of MDR-Ab using the variables that were significantly associated with the risk of MDR-Ab in multivariate analysis (tocilizumab therapy, ICU length of stay exceeding 11 days, and previous exposure to ceftriaxone). Each risk factor represented 1 point of the risk score. When adding previous exposure to ceftriaxone as a risk factor to the cohort of patients with no other risk factors, one or two other risk factors increase the proportion of patients with MDR-Ab in all cohorts. Moreover, the proportion of patients with MDR-Ab incrementally rose with the risk score (Table 6).

## 4. Discussion

Our retrospective study has confirmed MDR-Ab superinfection as a common complication of COVID-19 patients admitted to the ICU. In addition, exposure to ceftriaxone is an important modifiable risk factor of MDR-Ab superinfection.

### 4.1. *Acinetobacter* Superinfection in COVID-19

MDR-Ab is one of the most important causes of hospital-acquired infections in COVID-19 patients [8, 17]. MDR-Ab was the predominant superinfection in our cohort, affecting more than one third of ICU patients. Moreover, MDR-Ab was identified as the etiologic pathogen in more than three quarters of superinfections. A multicentric study by Pascale et al. identified *Acinetobacter* as the most common and clinically important cause of MDR superinfections in COVID-19 patients admitted to the ICU in Bologna, Italy [9]. Other authors have also reported multiple large outbreaks of MDR-Ab in ICUs during the COVID-19 pandemic [10–12]. MDR-Ab superinfection in COVID-19 patients significantly worsens the outcome of the disease and complicates its management [21, 23]. The ability to elude antimicrobial treatment and decontamination procedures allows *Acinetobacter* to cause large outbreaks, which are difficult to manage, especially in the setting of stretched hospital resources during the COVID-19 pandemic [10–12, 14–16]. Therefore, identification of modifiable risk factors of MDR-Ab superinfection in the COVID-19 setting is essential to improve the management of future waves of the COVID-19 pandemic.

### 4.2. *Acinetobacter* Superinfection in COVID-19 Patients and Antibiotic Exposure

We found that prior exposure to ceftriaxone is a strong independent risk factor of MDR-Ab infection. Ceftriaxone was the most prescribed antimicrobial drug in our study (38.9% of all patients). A recent study by Ceparano et al. identified carbapenem exposure but not cephalosporin exposure as a risk factor for *A. baumannii* isolation in COVID-19 ICU patients. In their study, the carbapenem exposure was much more prevalent (33.3% of all patients) than cephalosporin exposure (18.1% of all patients). This factor might have contributed to the results [27]. Based on univariate analyses, we found no association between meropenem exposure and the risk of MDR-Ab superinfection. However, most of our patients who were exposed to antimicrobials were treated by ceftriaxone and only 16% were treated by carbapenems. We hypothesise that the lack of association between MDR-Ab isolation and carbapenem exposure in our study is due to the low number of patients exposed to carbapenems. Study by Falcone et al. identified previous exposure to piperacillin-tazobactam as a risk factor of MDR superinfections in COVID-19 patients treated in the ICU. In contrast to our results, Falcone et al. found no significant association with prior ceftriaxone exposure. They even found that doxycycline therapy was associated with a lower risk of MDR superinfection. Doxycycline was the most prescribed antimicrobial drug in their study (47.6% of all patients). Ceftriaxone was prescribed in 46.6% and piperacillin-tazobactam in 14.3% of all patients in their study [26]. In our study, antibiotics other than ceftriaxone were used only rarely, so we did not include other antimicrobials in the multivariate analysis. In the study by Falcone et al., the most common isolated MDR pathogens were enterobacteria, not *Acinetobacter*. Nonfermenting Gram-negative rods were found in only up to 22% of patients with MDR superinfection in their study. This factor might also contribute to their different results [26]. In the prepandemic era, the risk of *Acinetobacter* infections in the ICU setting was found to be associated with previous exposure to broad-spectrum antimicrobials including cephalosporins and carbapenems [31–33]. According to this evidence, it is likely that exposure to any broad-spectrum antibiotic increases the risk of *Acinetobacter* colonisation in COVID-19 ICU patients; however, additional studies are needed to support this hypothesis [18].

### 4.3. Other Risk Factors of *Acinetobacter* Superinfection in COVID-19 Patients

Monoclonal antibodies against interleukin 6 (IL-6) like tocilizumab are recommended to treat COVID-19 patients requiring HFNO or mechanical ventilation because of its clear mortality benefits [18]. In our study, treatment with tocilizumab was associated with a higher risk for MDR-Ab isolation. Falcone et al. found an association between MDR infection and tocilizumab therapy in COVID-19 ICU patients. They also identified baricitinib therapy as a risk factor for MDR infections, which is another potent immunomodulator [26]. We were unable to evaluate the effect of baricitinib in our study because all of our ICU patients were treated with baricitinib. For the same reason, we were unable to assess the effect of corticosteroids. We found that ICU length of stay was associated with the risk of MDR-Ab isolation and infection in COVID-19 ICU patients. Falcone et al. also found this association [26]. In addition, they found an association between risk of MDR superinfection and mechanical ventilation [26]. In our study, there was a significantly higher portion of mechanically ventilated patients among those with MDR-Ab superinfection; however, the association was not present in multivariate analysis. Invasive procedures, orotracheal intubation, mechanical ventilation, and the use of corticosteroids and immunomodulators were identified as possible risk factors for MDR bacterial superinfections in the ICU environment in the prepandemic era [31–33]. The presence of risk factors in our study had a cumulative effect on the proportion of patients with MDR-Ab. In the cohort of patients with no risk factors, the prevalence of MDR-Ab superinfection was 12%. However, in the cohort of patients treated with tocilizumab, exposed to ceftriaxone, and with an ICU stay exceeding 11 days, the prevalence of MDR-Ab superinfection was 85%.

### 4.4. Clinical Relevance and Implications

Clinical guidelines for treatment of critically ill COVID-19 patients state that there are insufficient data to recommend for or against the use of empiric broad-spectrum antimicrobial therapy in the absence of another indication [18]. However, there is growing evidence that empiric antibiotic therapy might be harmful even in critically ill COVID-19 patients because it is associated with the risk of MDR bacterial superinfection. Previous studies have found that risk of MDR isolation in COVID-19 patients is associated with broad-spectrum antibiotics like piperacillin-tazobactam and carbapenems, but not with narrower spectrum antibiotics like cephalosporins and aminopenicillins [26, 27]. We identified ceftriaxone as an independent and potent risk factor for MDR-Ab superinfection in the setting of COVID-19 ICU patients. The risk of MDR-Ab superinfection development attributed to ceftriaxone exposure in our study is considerably high. The OR for ceftriaxone exposure in the binary logistic regression model was 4.1 (95% CI 1.4–11.9). In our cohort of patients, exposure to ceftriaxone in addition to another risk factor such as tocilizumab therapy or ICU length of stay exceeding 11 days resulted in a significant increase in the proportion of patients with MDR-Ab superinfection (from 23.8% to 68.8%). Considering these findings, we suggest that broad-spectrum antimicrobial treatment in general increases the risk of MDR superinfection and that antibiotic treatment in COVID-19 patients should be avoided unless there is a clear clinical indication (established presence of bacterial superinfection), especially in the presence of other risk factors, like exposure to tocilizumab or a predicted long ICU stay. True bacterial superinfections in COVID-19 patients are uncommon, and most patients do not require antibiotic therapy. Nevertheless, the use of antibiotics has increased sharply during the COVID-19 pandemic and there has been significant overuse of empiric antimicrobial therapy in COVID-19 patients [4, 24, 25]. Overuse of antibiotics promotes the development of antibiotic resistance and MDR superinfections and needs to be properly addressed in hospital antibiotic stewardship programmes, even during COVID-19 pandemic waves. We also suggest that this issue be addressed in more detail in COVID-19 treatment guidelines.

## 5. Strengths and Limitations

This study has several strengths. First, the sampling for biological material for cultivation was performed on a regular basis in all patients; therefore, variance in sampling frequency did not affect the results. Second, all patients were hospitalised in the same ICU during a single wave of the COVID-19 pandemic caused predominantly by the delta variant; hence, the study population is homogenous. The delta variant occurred as dominant variant in Europe in July 2021 and remained dominant in Slovakia until January 2022 when it was outcompeted by omicron variant [33–35]. Our study also has some limitations. The first limitation is its retrospective design. Retrospective studies are inheritably more prone to biases. We tried to minimize the risk of bias by including all patients to mitigate the possible risk of selection bias, and we performed multivariate analysis to mitigate the effects of possible confounders. Second, the sample size is relatively small, and the CIs of some significant predictors are quite broad. This factor is also probably responsible for some of the predictors of MDR-Ab infection showing a trend but not a significant association. Third, we were unable to assess the association between MDR-Ab risk and exposure to other antibiotics because most of our patients were treated with ceftriaxone and other antimicrobials were used rarely. Therefore, additional studies are needed to describe in more detail the risk associated with antibiotic exposure and MDR superinfections in COVID-19 patients.

## 6. Conclusions

Exposure to ceftriaxone significantly increases the risk for MDR-Ab superinfection in COVID-19 patients admitted to the ICU. Our findings suggest that antimicrobial treatment in the COVID-19 ICU should be restricted to patients with clinically apparent bacterial infection. However, additional studies are needed to evaluate the role of antimicrobial therapy in COVID-19 critical care.

## Figures and Tables

**Table 1:** The prevalence of MDR bacteria in our study population.

Class of MDR bacteria	Prevalence, *n* (%)
*Acinetobacter*	37 (41.1%)
*Pseudomonas aeruginosa*	12 (13.33%)
*Stenotrophomonas maltophilia*	5 (5.56%)
Enterococci	7 (7.78%)
Enterobacteria	9 (10%)
Overall MDR bacteria prevalence	43 (47.78%)
MDR: multi drug resistant; *n*: number of subjects	

**Table 2:** The resistance profile of *Acinetobacter* isolates. If more than one strain was isolated in one patient, the more resistant strain was included in the analysis.

Antimicrobial agent	*n* (%)
Ampicilin	37 (100%)
Ampicilins-sulbactam	23 (62%)
Piperacilin	37 (100%)
Piperacilin-tazobactam	36 (97%)
Cefotaxime	33 (89%)
Ceftazidime	25 (67%)
Cefoperason-sulbactam	36 (97%)
Cefepim	23 (62%)
Aztreonam	37 (100%)
Imipenem	20 (54%)
Meropenem	20 (54%)
Gentamycin	20 (54%)
Amikacine	20 (54%)
Ciprofloxcin	37 (100%)
Colimycine	1 (3%)
Trimetoprim-sulfametoxazol	21 (57%)

*n*: number of subjects.

**Table 3:** Basic characteristics of the patients.

Variable	MDR-Ab infection	No MDR-Ab infection
Quantitative variables	Median (25th percentile, 75th percentile)	
Age (years)	66 (56, 70)	64 (50, 70)
C-reactive protein (mg/L)	130 (76, 170)	112 (65, 149)
Glomerular filtration rate (ml/s)	1.32 (0.87, 1.59)	1.24 (0.96, 1.48)
Neutrophil count (×1000 cells/ml)	7.45 (4.85, 13.29)	7.69 (4.46, 12.04)
Lymphocyte count (×1000 cells/ml)	0.74 (0.48, 1.34)	0.79 (0.54, 1.36)
CD4+ lymphocyte count (cells/ml)	285 (180, 462)	300 (160, 430)
Length of ICU stay	13 (6, 18)^*∗*^	6 (4, 9)

Categorical variables	*n*/*n* total (%)	
Diabetes mellitus	13/33 (39.39%)	19/57 (33.33%)
Obesity	19/33 (57.58%)	38/57 (49.12%)
Male gender	21/33 (63.63%)	36/57 (63.16%)
Tocilizumab therapy	13/33 (39.39%)^*∗*^	7/57 (12.28%)
Previous exposure to ceftriaxone	20/33 (60.60%)^*∗*^	17/57 (29.82%)
Previous exposure to meropenem	6/33 (18.18%)	9/57 (15.78%)
Mechanical ventilation	20/33 (60.60%)^*∗*^	22/57 (38.60%)
Death	17/33 (51.52%)	21/57 (36.84%)

^*∗*^ *p* < * *0.05 in univariate analysis. ICU: intensive care unit; MDR-Ab: multidrug-resistant *Acinetobacter*.

**Table 4:** Univariate analysis of the association between possible risk factors and the risk of MDR-Ab isolation.

Variable	Odds ratio (95% confidence interval)	*P*
Age (years)	1.01 (0.97–1.04)	0.664
Length of ICU stay exceeding 11 days	2.20 (1.27–3.81)	0.001
Diabetes mellitus	1.10 (0.79–1.53)	0.649
Obesity	0.79 (0.46–1.35)	0.497
Tocilizumab therapy	1.45 (1.08–1.94)	0.004
Mechanical ventilation	1.56 (0.97–2.50)	0.051
Previous ceftriaxone exposure	1.83 (1.16–2.87)	0.004
Previous meropenem exposure	1.03 (0.85–1.25)	0.559
C-reactive protein (mg/L)	1.00 (0.99–1.01)	0.604
Glomerular filtration rate (ml/s)	1.17 (0.42–3.30)	0.329
Neutrophil count (×1000 cells/ml)	1.06 (0.98–1.14)	0.683
Lymphocyte count (×1000 cells/ml)	1.07 (0.77–1.47)	0.573
CD4+ lymphocyte count (cells/ml)	1.00 (1.00–1.00)	0.732

ICU: intensive care unit; MDR-Ab: multidrug-resistant *Acinetobacter*; *P*: probability.

**Table 5:** Multivariate analysis of association of possible risk factors with the risk of MDR-Ab isolation.

Variable	Odds ratio (95% confidence interval)	Beta	*P*
Previous ceftriaxone exposure	4.11 (1.42–11.91)	1.41	<0.05
Tocilizumab therapy	4.47 (1.33–15.03)	1.50	<0.05
Mechanical ventilation	1.80 (0.64–5.09)	0.59	0.26
ICU length of stay exceeding 11 days	3.71 (1.33–10.32)	1.31	<0.05

ICU: intensive care unit; MDR-Ab: multidrug-resistant *Acinetobacter*; *P*: probability. Associations between MDR-Ab isolation and infection and age, diabetes mellitus, ICU length of stay exceeding 11 day, mechanical ventilation, obesity, previous ceftriaxone exposure, previous meropenem exposure, tocilizumab therapy, C-reactive protein, glomerular filtration rate, neutrophil count, lymphocyte count, and CD4+ lymphocyte count were assessed using a multivariate binary logistic regression model with a forward stepwise procedure, entering all variables with *P* < 0.1 in univariate analysis. Statistical significance was established at *P* < 0.05. Only variables such as ICU length of stay exceeding 11 day, mechanical ventilation, previous ceftriaxone exposure, and tocilizumab therapy were included in the final model. Other variables were excluded because they were found to be insignificant in the forward stepwise procedure.

**Table 6:** The effects of risk factors and their accumulation on the proportion of patients with multidrug-resistant *Acinetobacter* superinfection.

Risk points	No previous ceftriaxone exposure *n*/*n* total (%)	Previous ceftriaxone exposure *n*/*n*total (%)
0	3/25 (12.0%)	3/13 (23.1%)
1	5/21 (23.8%)	11/16 (68.8%)^*∗*^
2	5/8 (62.5%)	6/7 (85.7%)

Patients received 1 point for the presence of tocilizumab therapy and 1 point for an intensive care unit length of stay exceeding 11 days. *n*: number of patients with multidrug-resistant *Acinetobacter* superinfection. ^*∗*^*P* < * *0.05 (chi-square test).

## Data Availability

The dataset used and analysed during this study is available from the corresponding author upon reasonable request.

## Abbreviations

CI: Confidence interval
COVID-19: Coronavirus disease 2019
HFNO: High-flow nasal oxygen
ICU: Intensive care unit
MDR: Multidrug-resistance
MDR-Ab: Multidrug-resistant *Acinetobacter*
OR: Odds ratio
*P*: Probability
SARS-CoV-2: Severe acute respiratory distress syndrome coronavirus 2.
